# The sodium channel β1 subunit mediates outgrowth of neurite-like processes on breast cancer cells and promotes tumour growth and metastasis

**DOI:** 10.1002/ijc.28890

**Published:** 2014-04-12

**Authors:** Michaela Nelson, Rebecca Millican-Slater, Lorna C Forrest, William J Brackenbury

**Affiliations:** 1 Department of Biology, University of YorkHeslington, York, YO10 5DD, United Kingdom; 2 Department of Histopathology, St James's University HospitalLeeds, LS9 7TF, United Kingdom

**Keywords:** adhesion, breast cancer, fyn kinase, metastasis, voltage-gated Na^+^ channel

## Abstract

Voltage-gated Na^+^ channels (VGSCs) are heteromeric proteins composed of pore-forming α subunits and smaller β subunits. The β subunits are multifunctional channel modulators and are members of the immunoglobulin superfamily of cell adhesion molecules (CAMs). β1, encoded by *SCN1B*, is best characterized in the central nervous system (CNS), where it plays a critical role in regulating electrical excitability, neurite outgrowth and migration during development. β1 is also expressed in breast cancer (BCa) cell lines, where it regulates adhesion and migration *in vitro*. In the present study, we found that *SCN1B* mRNA/β1 protein were up-regulated in BCa specimens, compared with normal breast tissue. β1 upregulation substantially increased tumour growth and metastasis in a xenograft model of BCa. β1 over-expression also increased vascularization and reduced apoptosis in the primary tumours, and β1 over-expressing tumour cells had an elongate morphology. *In vitro*, β1 potentiated outgrowth of processes from BCa cells co-cultured with fibroblasts, *via trans*-homophilic adhesion. β1-mediated process outgrowth in BCa cells required the presence and activity of fyn kinase, and Na^+^ current, thus replicating the mechanism by which β1 regulates neurite outgrowth in CNS neurons. We conclude that when present in breast tumours, β1 enhances pathological growth and cellular dissemination. This study is the first demonstration of a functional role for β1 in tumour growth and metastasis *in vivo*. We propose that β1 warrants further study as a potential biomarker and targeting β1-mediated adhesion interactions may have value as a novel anti-cancer therapy.

Although the death rate from breast cancer (BCa) is falling in many countries, it is still the leading cause of cancer-related deaths in women, due to metastasis.^1^,^2^ To metastasize, tumour cells undergo a complex sequence of events, including adhesion/detachment, migration, and invasion. Given that treatment options for metastatic BCa are mainly restricted to palliation, it is necessary to better understand the mechanism(s) involved in order to identify new targets and develop new therapies.^3^

Voltage-gated Na^+^ channels (VGSCs) contain one pore-forming α subunit with smaller β subunits.^4^ There are nine α subunits, Na_v_1.1-Na_v_1.9, and four β subunits, β1-β4. The β subunits are members of the immunoglobulin superfamily of cell adhesion molecules (CAMs). They modulate channel gating, and can function as CAMs both in the presence and absence of α subunits.^5^ They are substrates for secretase cleavage, releasing soluble intracellular domains that may regulate gene expression.^6^ The β1 subunit (gene: *SCN1B*) participates in *trans*-homophilic adhesion, resulting in cellular aggregation and cytoskeleton recruitment.^7^,^8^ β1 also interacts heterophilically with other CAMs, including β2, contactin, neurofascins, NrCAM, N-cadherin^9^–^12^ and the extracellular matrix protein, tenascin-R.^13^ β1 mediates neurite outgrowth by a *trans*-homophilic adhesion mechanism that requires fyn kinase, contactin and γ-secretase activity.^5^,^14^,^15^ β1 plays a critical role during central nervous system (CNS) development, regulating electrical excitability, proliferation, fasciculation, pathfinding and migration.^15^–^17^

VGSCs are widely expressed in cancers, and contribute to cellular behaviours associated with metastasis.^18^,^19^ In BCa, the predominant α subunit, Na_v_1.5 (gene: *SCN5A*), is expressed in MDA-MB-231 cells, where Na^+^ current potentiates invasion by enhancing cysteine cathepsin activity.^20^–^22^ *SCN5A* is up-regulated in tumours, associating with recurrence, metastasis and reduced survival.^20^,^23^ β1 is the predominant β subunit in MCF-7 cells, where it enhances cell-substrate adhesion, but slows transwell migration.^24^ Over-expression of β1 in MDA-MB-231 cells increases cell-cell adhesion and Na^+^ current.^24^ Both α and β1 subunits are expressed in lamellipodia of MCF-7 and MDA-MB-231 cells, suggesting that their expression and function are not mutually exclusive.^23^ Thus, VGSC α and β subunits appear to play complex, dynamic roles in metastatic BCa cells. However, the functional significance of β1-dependent adhesion, and its contribution to tumour growth and metastasis, are unknown.

Our aim here was to study the involvement of β1 in BCa progression *in vivo*. We show that *SCN1B* mRNA/β1 protein are upregulated in BCa specimens, compared with normal breast tissue. Up-regulation of β1 potentiates tumour growth and metastasis *in vivo*. In addition, β1 increases process outgrowth on BCa cells *via* a *trans*-homophilic adhesion mechanism that requires fyn kinase and Na^+^ current. We propose that β1 enhances metastatic behaviour of BCa cells by recapitulating mechanism(s) that are critical for neuronal migration during CNS development. These findings suggest that β1 warrants further study as a potential biomarker/therapeutic target.

## Methods

### *In silico* analysis

*SCN1B* expression in microarrays was studied using Oncomine.^25^ Meta-analysis of correlations between *SCN1B* expression and histoclinical characteristics across multiple datasets was as described.^26^ Datasets, patients, specimen characteristics and assay methods are detailed/referenced at http://www.oncomine.org.

### Cell culture

Molecular identity of all BCa cell lines was confirmed by short tandem repeat analysis. All cell lines were grown in Dulbecco's modified eagle medium (DMEM) supplemented with 5% fetal bovine serum and 4 m*M* l-glutamine.^23^ BT474 and SKBR3 cells were a gift from J. Rae, University of Michigan. MCF-7 and MDA-MB-231 cells were a gift from M. Djamgoz, Imperial College London. “Control” MDA-MB-231 cells stably expressing enhanced green fluorescent protein (GFP) or MDA-MB-231 cells over-expressing β1-GFP C-terminal fusion^24^ (hereafter called “MDA-MB-231-β1” cells) were maintained in medium containing selective antibiotics. MCF-10A cells were a gift from N. Maitland, University of York. R1610 Chinese hamster lung (CHL) fibroblasts and CHL fibroblasts stably expressing β1 were gifts from L. Isom, University of Michigan. MDA-MB-231-GFP and MDA-MB-231 β1-GFP cells were stably transduced with recombinant lentivirus for firefly luciferase (AMS Biotechnology). For experiments using estrogen, MCF-7 cells were maintained in phenol red-free DMEM supplemented with 5% charcoal-stripped fetal bovine serum and 4 m*M* L-glutamine. Cells were confirmed as mycoplasma-free using the DAPI method.

### Pharmacology

Tetrodotoxin (TTX) was diluted in culture medium to 30 µ*M*. Staurosporine, PP2, estradiol and fulvestrant were prepared as stocks in DMSO and then diluted in culture medium to 10 n*M*−30 µ*M*. In assays that exceeded 24 h, treatments were replaced daily. The effect of TTX on invasion was determined using Matrigel assays.^23^ The effect of staurosporine on apoptosis was determined using DeadEnd fluorometric TUNEL assays (Promega). The effect of PP2 on cell viability and proliferation was determined using trypan blue and MTT assays.^23^

### RNA isolation and RT-qPCR

RNA extraction and cDNA synthesis were as described.^27^ QPCR was carried out using triplicate 12-µl reactions containing 20ng cDNA. Amplification conditions were: 95°C for 30 s followed by 35 cycles of 95°C for 5 s and 60°C for 10 s on a Bio-Rad thermal cycler. Relative gene expression was quantitated using the comparative *C*_T_ method. Primers are in Supporting Information Table S1.

### Patient tissue samples

The study cohort contained tissue samples from 66 BCa cases obtained from the Breast Cancer Campaign Tissue Bank under tissue request number TR000017. Patients provided consent to the Breast Cancer Campaign Tissue Bank for their tissues to be used for research. The samples came from women aged 28–89 years, who were diagnosed between February 1992 and February 2012. For 40 cases (60%), tumour samples came with matched surrounding normal breast tissue. Clinical and histopathological data were available for all cases. Immunohistochemistry was performed on 5µm-thick sections using the EnVision+ System-HRP kit (Dako). Sections were deparaffinized in Histo-Clear (National Diagnostics) followed by antigen retrieval at 95°C for 30 min in Target Retrieval Solution (Dako). Sections were incubated with anti-β1 antibody (1:25; Abgent) for 30 mins and counterstained with dilute Mayer's hematoxylin and mounted in Faramount medium (Dako). Slides were scanned at 40× using an Aperio ScanScope. β1 immunoreactivity was scored by two independent investigators (WJB and RMS, a breast histopathologist) using the Allred method.^28^ Briefly, the proportion of β1-positive cells was given a score (none: 0; <1/100: 1; 1/100 to 1/10: 2; 1/10 to 1/3: 3; 1/3 to 2/3: 4; >2/3: 5), followed by the intensity of staining (none: 0; weak: 1; intermediate: 2; strong: 3). For each section, the proportion and intensity scores were summed to give a total score (0–8). A score of 0–4 was considered “low” and 5–8 was considered “high.” Scoring was performed without prior knowledge of outcome data. Experiments were approved by the University of York Ethical Review Process.

### Western blotting

SDS-PAGE was performed as described.^15^,^29^ The following antibodies were used: rabbit anti-β1 (1:100; Abgent), mouse anti-fyn (1:1,000; BioLegend), mouse anti-CD44 (1:1,000; AbD Serotec); rabbit anti-E-cadherin (1:1,000; Cell Signaling Technology); mouse anti-β-actin (1:30,000; Proteintech); and mouse anti-α-tubulin (1:10,000; Sigma). Signals were quantified using ImageJ software. α-tubulin was used as loading control.

### Orthotopic breast tumour model

All animal procedures were carried out after approval by the University of York Ethical Review Process and under authority of a UK Home Office Project Licence. Six-week-old female *Rag2*^−/−^ *Il2rg*^−/−^ mice (mean weight: 16.6 ± 0.2 g) were obtained from the Yorkshire Cancer Research Unit, University of York. Mice (4–5 per specific pathogen free cage) were selected at random for surgery. A 1 × 10^6^ control MDA-MB-231-GFP or MDA-MB-231-β1-GFP cells expressing luciferase were suspended in Matrigel (20% v/v in saline) and injected into the left and right inguinal mammary fat pad of each animal whilst under isoflurane anaesthesia. A total of 13 mice were used (six injected with control cells and seven with β1 cells) across three independent replicated experiments. Tumour growth was monitored weekly by bioluminescence imaging. Mice were given intraperitoneal injection of D-luciferin in PBS (150 mg kg^−1^) and bioluminescence was visualized 10 min later under isoflurane anaesthesia using an IVIS100 system (PerkinElmer). Bioluminescence from tumours was quantified within manually defined regions of interest using Living Image software (PerkinElmer) and expressed as photon flux. To quantify bioluminescence at sites of metastasis, mice were euthanized ∼10 min after injection with D-luciferin, primary tumours were removed and internal organs were exposed by dissection. Bioluminescence was measured from the entire mouse and then individual organs were removed for separate imaging. Measurements of the length and width of each tumour (in mm) were taken from mice daily with callipers. Tumour volume was calculated as 0.5 × (length × width^2^). Mice were euthanized when primary tumours reached 10% of starting body weight, or at the first sign of discomfort from metastatic burden. Tumours and organ sites of metastasis were fixed in 4% paraformaldehyde and frozen.^14^

### Immunohistochemistry (IHC) and immunocytochemistry (ICC)

For H&E staining, sections were stained with Gill's hematoxylin and eosin Y and then scanned at 20X using a Zeiss AxioScan.Z1 slide scanner. The following primary antibodies were used for IHC/ICC^15^: rabbit anti-Ki67 (1:5,000; Abcam); rabbit anti-activated caspase-3 (1:200; R&D Systems); rabbit anti-CD31 (Santa Cruz Biotechnology); mouse anti-skeletal myosin (1:400; Sigma); rabbit anti-β1 (1:200; Abgent); mouse anti-fyn (1:100; BioLegend); mouse anti-CD44 (1:100; AbD Serotec); rabbit anti-E-cadherin (1:200; Cell Signaling Technology); mouse anti-human nuclear antigen (HNA; 1:100; Millipore). Secondary antibodies were Alexa-568-conjugated goat anti mouse/rabbit, unless stated otherwise (1:500; Invitrogen). Samples were mounted in Prolong Gold with DAPI (Invitrogen). Some samples were stained with Alexa-633-phalloidin (1:25; Invitrogen).^23^ Samples were viewed using 20× objectives on a Nikon Eclipse TE200 fluorescent microscope, or Zeiss Axio Observer.Z1 microscope with LSM 710 confocal laser scanner.

### Image analysis

Images were exported into ImageJ for processing. Confocal Z-series projections were flattened using the maximum signal. Brightness/contrast was adjusted using the ImageJ “Auto” function. ICC colocalization was evaluated using ImageJ. Intensity correlation analysis (ICA) was performed on individual cells delineated with the freehand selection tool, and for each cell, the intensity correlation quotient (ICQ) was computed. For signal intensities that vary together, indicating colocalization, 0 < ICQ ≤ 0.5, whereas for segregated staining, −0.5 ≤ ICQ < 0.^30^ Measurements were from 20 cells per line.

The following measurements were made on IHC sections, for three mice per treatment group:

- Density of Ki67^+^ or activated caspase-3^+^ cells^17^: the number of Ki67^+^ nuclei or active caspase-3^+^ cells was counted per 20X field of view.
- Tumour vascularity^31^: the number of CD31^+^ vessels was counted per 20X field of view.
- Metastasis to liver/lungs/spleen^32^: the number of GFP^+^ metastatic foci was counted per 20X field of view.
- Length of tumour cell processes and muscle fibers: The longest visible process on cancer cells and the total length of individual muscle fibers within 20× fields of view was measured using the freeform line function in ImageJ.

### VEGF ELISA

Cells were cultured in 24-well plates (5 × 10^4^/well). After 1, 2, and 3 days, culture medium was removed from individual wells and stored at −20°C until analysis. VEGF secretion was determined by ELISA (Promega). Measurements were obtained from duplicate wells from three repeat experiments.

### Process outgrowth assay

Process outgrowth assays were based on Ref.^14^. Parental CHL fibroblasts or CHL fibroblasts expressing β1 were grown to confluence on 13mm diameter coverslips. Freshly dissociated BCa cells were plated (2 × 10^4^ cells/well) on top of the monolayers and allowed to grow for 24–48 h. Cultures were fixed in 4% paraformaldehyde and the cancer cells were visualized with anti-GFP (1:1,000; NeuroMab), or anti-cytokeratin 18 (1:500; BioLegend) followed by Alexa-568-conjugated goat anti-mouse (1:500). Images were acquired using a Nikon Eclipse TE200 fluorescent microscope with 40× objective. The longest process on each of the first 50 randomly selected, isolated cancer cells was measured using ImageJ. Measurements were obtained from three independent experiments.

### Deletion of β1 Ig domain

The Ig domain of β1 (amino acids 40–124) in pEGFPN1^24^ was deleted using the Phusion site-directed mutagenesis kit (Thermo Scientific). The β1Δ_40–124_-GFP construct was sequenced, and then transfected into MDA-MB-231 cells using Fugene (Roche).^24^

### RNA interference

SiGENOME SMARTpool siRNA targeting *FYN* and siGENOME Non-Targeting siRNA Pool #1 (Dharmacon) were used at 50 n*M*. Transfection was performed using Dharmafect 1 reagent. Transfection efficiency was confirmed to be ≥90% using siGENOME positive control targeting *GAPDH* (Supporting Information Fig. S5*c*). RNA extraction and process outgrowth assay were performed 96h after transfection.

### Data analysis

Data are mean and SEM unless stated otherwise. Statistical analysis was performed using GraphPad Prism. Matrix data were plotted using Matrix2png software.^33^ Statistical significance was determined with *t* tests or Mann–Whitney tests, and multiple comparisons were made using ANOVA and Tukey *post hoc* tests, unless stated otherwise. *p* values computed by Oncomine were corrected for multiple comparisons by Bonferroni method. Correlation between *ESR1* and *SCN1B* expression was determined using Pearson's *r* test. Association between categorical classification criteria was determined with Fisher's exact test, or *χ*^2^ test. For meta-analysis of association between *SCN1B* expression and histoclinical characteristics across multiple datasets, the binomial test was used.^26^ The binomial test *p* value indicates whether or not one criterion was associated with another in the observed number of datasets by chance, given the number of datasets studied. Kaplan–Meier curves for survival were compared by log-rank tests. Percent survival and hazard ratios are presented with 95% confidence intervals. Results were considered significant at *p* < 0.05.

## Results

### β1 mRNA and protein are present in breast tumours

**Figure 1:**
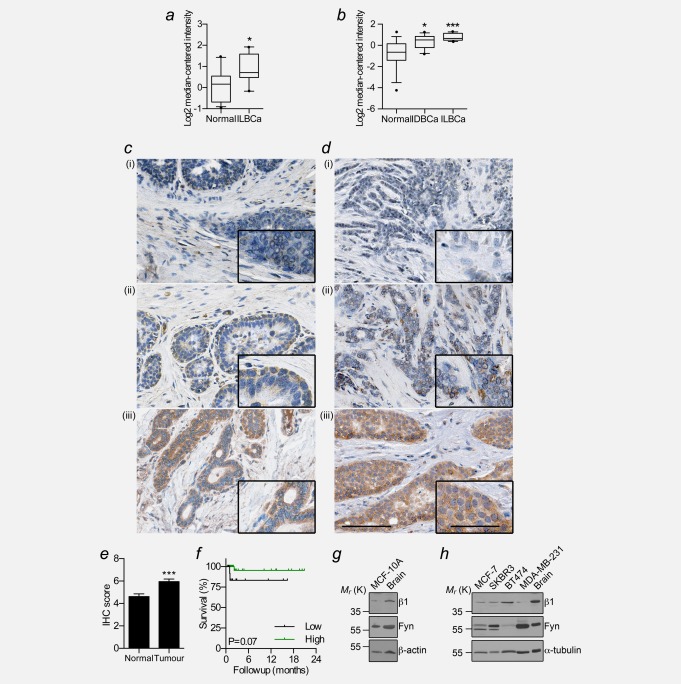
β1 mRNA and protein expression in breast cancer. (*a*) Expression of *SCN1B* mRNA in invasive lobular breast carcinoma (ILBCa) *vs*. normal in the Radvanyi Breast dataset (*n* = 15). (*b*) Expression of *SCN1B* in invasive ductal breast cancer (IDBCa) and ILBCa in the Turashvili Breast dataset (*n* = 30). Box plot dots, maximum and minimum values; whiskers, 90th and 10th percentile values; and horizontal lines, 75th, 50th, and 25th percentile values. (*c*) Representative images of non-cancer breast tissue and (*d*) breast tumour tissue in which β1 was (i) absent, (ii) weakly positive, and (iii) strongly positive. Scale bar, 100 µm. Insets, higher magnification images of tumour cells, scale bar, 50µm. (*e*) Mean Allred score for normal breast and tumour samples. Data are mean ± SEM (*n* = 66). (*f*) Kaplan–Meier analysis comparing BCa-specific survival of those with “low” (score <5) *vs*. “high” (score ≥ 5) β1 expression (*n* = 62). (*g*) Western blot of β1 and fyn in the MCF-10A non-cancer mammary epithelial cell line. (*h*) Western blot of β1 and fyn across a panel of BCa cell lines. Positive control = rat brain lysate. *p < 0.05; **p < 0.01; ***p < 0.001.

We have previously shown that β1 mRNA/protein are expressed in BCa cell lines.^24^ Here, we used Oncomine to study the expression of *SCN1B* mRNA in normal breast and BCa specimens across multiple microarrays. *SCN1B* was expressed at a significantly higher level in BCa compared with normal breast in two out of eight datasets in which differential data were available (1.7-fold, *p* < 0.05; and >2.2-fold, *p* < 0.05; Figs. 1*a* and 1*b*). We next performed a meta-analysis to investigate whether *SCN1B* expression correlates with histoclinical characteristics across multiple datasets. High *SCN1B* expression associated with ER status in 8/21 (38.1%) of datasets (*p* < 0.0001; Supporting Information Table S2; Figs. S1*a* and S1*b*). There was no significant association between *SCN1B* and age, pathological tumour size, grade, recurrence, progesterone receptor, or HER2 status across the same datasets. Up-regulation of *SCN1B* expression in ER^+^ tumours correlated with several genomic neighbours on chromosome 19q (Supporting Information Fig. S1*c*).^26^ However, mRNA levels of the two *SCN1B* splice variants, β1 and β1B,^34^ which are both expressed across a panel of BCa cell lines (Supporting Information Figs. S1*d* and S1*e*), were unchanged in MCF-7 cells following treatment with estrogen or fulvestrant (Supporting Information Fig. S1*f*), suggesting that *SCN1B* is not estrogen-regulated.

We next studied the expression of β1 at protein level in human tissue samples by IHC. β1 immunoreactivity was mainly in the cytoplasm of epithelial and carcinoma cells, with variable expression at the plasma membrane ( Figs. 1*c* and 1*d*). This pattern of expression is consistent with previous observations in neurons and cancer cell lines.^14^,^16^,^23^ β1 expression was significantly higher in tumour than normal, non-cancer breast tissue samples (*p* < 0.001; Fig. 1*e*). Of the cases where tumour had matched surrounding non-cancer tissue, 27 (68%) had higher β1 in tumour than non-cancer tissue, 7 (17%) had the same level of β1 in tumour and non-cancer tissue, and 6 (15%) had lower expression in tumour than non-cancer tissue. β1 expression in the tumour did not correlate with age, ER status, grade, menopausal status, or node status (Supporting Information Table S3). Similarly, there was no relationship with BCa-specific survival ( Fig. 1*f*, Supporting Information Table S3). In agreement with the IHC data, β1 was also expressed at protein level in the non-cancer mammary epithelial MCF-10A cell line and across a panel of BCa cell lines (Figs. 1*g* and 1*h*). Together, these data suggest that β1 may be up-regulated in a unique subset of breast cancers at mRNA and protein level.

### β1 promotes tumour growth and vascularization

**Figure 2:**
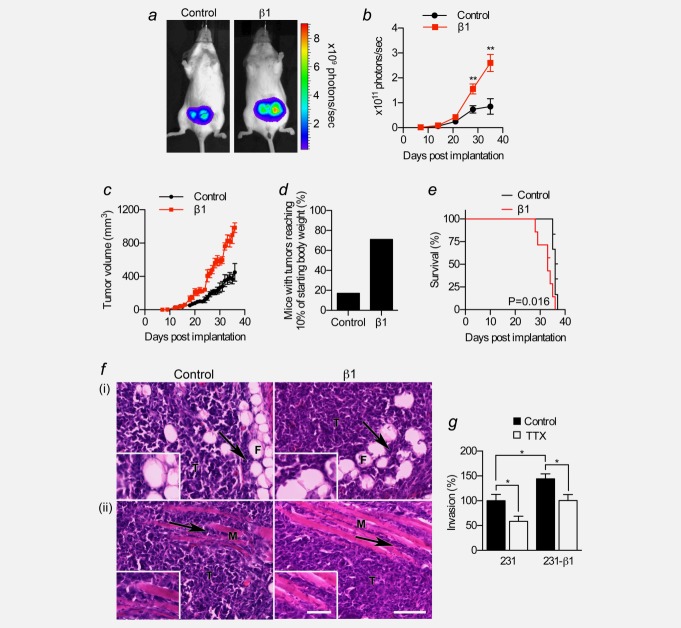
Effect of β1 over-expression on breast tumour growth *in vivo*. MDA-MB-231 (“Control”) and MDA-MB-231-β1 (“β1”) cells were implanted into the inguinal mammary fat pads of female *Rag2*^−/−^ *Il2rg*^−/−^ mice. (*a*) Representative bioluminescent images of mice bearing control and β1 tumours, 4 weeks after implantation. (*b*) Bioluminescence measured from primary tumours on the indicated days post-implantation (*n* ≥ 12). Data are mean ± SEM; **p < 0.01. (*c*) Calculated volume derived from calliper measurement of primary tumours over the same period (*n* ≥ 12). (*d*) Percentage of mice whose primary tumour burden reached 10% of starting body weight within the 5-week tumour growth period is shown for control and β1 tumours. (*e*) Kaplan–Meier analysis comparing overall survival of mice bearing control and β1 tumours (*n* = 13). (*f*) Images of control and β1 tumour tissue sections stained with H&E showing (i) mammary fat pad and (ii) skeletal muscle invasion. Arrows, infiltration of tumour cells (T) into fibroadipose tissue (F) or skeletal muscle fibers (M). Scale bar, 100 µm. Insets, higher magnification images of invading tumour cells, scale bar, 50 µm. (G) Invasion of control MDA-MB-231 and MDA-MB-231-β1 cells ± TTX (30 µM) for 48 hr (*n* = 12; *p < 0.05; Neuman–Keuls test).

We next investigated the role of β1 in tumour growth and metastasis. We orthotopically implanted luciferase-expressing MDA-MB-231 or MDA-MB-231-β1 cells into the mammary fat pads of female *Rag2*^−/−^ *Il2rg*^−/−^ mice and monitored tumour growth by bioluminescent imaging. We chose this model because MDA-MB-231 cells rapidly form palpable tumours following orthotopic implantation, and the cells readily metastasise. MDA-MB-231 cells express very low endogenous β1 (Supporting Information Fig. S2*a*). By contrast, MDA-MB-231-β1 cells over-express β1-GFP by >40-fold relative to endogenous β1 (Supporting Information Figs. S2*a* and S2*b*).^24^ Over-expression of β1 had no effect on expression of CD44 or E-cadherin (Supporting Information Figs. S2*c*–S2*e*). Importantly, luciferase activity and GFP expression were very similar in both cell lines (Supporting Information Figs. S3*a*–S3*d*). Photon flux from MDA-MB-231-β1 tumours increased faster than control MDA-MB-231 tumours, becoming statistically significant after 4 weeks ( Figs. 2*a* and 2*b*). To confirm the bioluminescent imaging data, we also analyzed tumour growth by daily calliper measurement. The volume of MDA-MB-231-β1 tumours increased more rapidly than MDA-MB-231 tumours, closely agreeing with the bioluminescent data ( Fig. 2*c*). During the 5-week study period, MDA-MB-231-β1 primary tumour burden reached 10% of starting body weight in 71% of mice, compared with only 17% for control tumours ( Fig. 2*d*). The survival of mice bearing MDA-MB-231-β1 tumours was significantly reduced compared to those bearing control tumours (*p* < 0.05; hazard ratio = 6.3 [1.4–27.8]; Fig. 2*e*). These data demonstrate that over-expression of β1 enhanced the growth of breast tumours, thus reducing survival.

**Figure 3:**
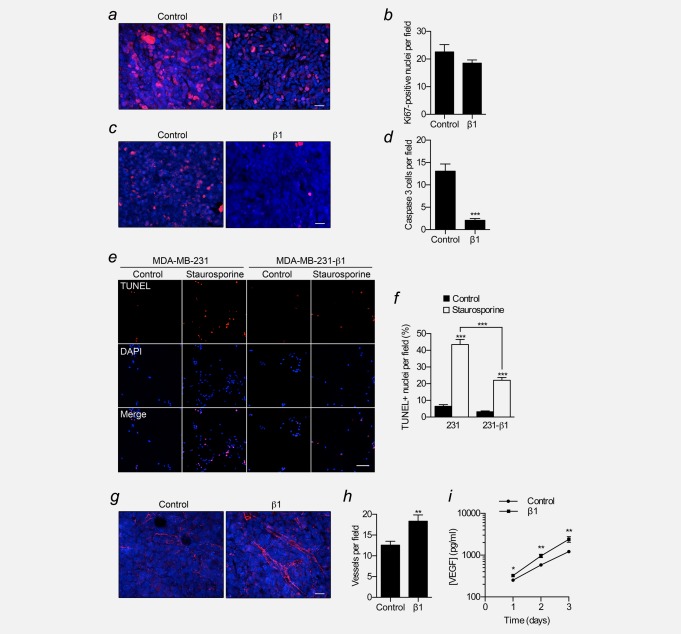
Effect of β1 upregulation on proliferation, apoptosis and angiogenesis. (*a*) Control and β1 tumour sections stained with anti-Ki67 (red) and DAPI (blue). Scale bar, 20 µm. (*b*) Ki67-positive nuclei per field of view for control and β1 tumours (*n* = 30). (*c*) Control and β1 tumour sections stained with anti-activated caspase-3 (red) and DAPI (blue). Scale bar, 20 µm. (*d*) Activated caspase-3-positive cells per field of view for control and β1 tumours (*n* = 30). (*e*) Images of control and MDA-MB-231-β1 cells treated for 24 hr with/without 0.5 µ*M* staurosporine, analyzed by TUNEL assay (red), counterstained with DAPI (blue). Scale bar, 100 µm. (*f*) Proportion (%) of TUNEL-positive nuclei per field of view (*n* = 60). (*g*) Blood vessels stained with endothelial marker CD31 (red) and DAPI (blue) in control and β1 tumour sections. Scale bar, 20 µm. (*h*) CD31-positive blood vessels per field of view for control and β1 tumours (*n* = 30). (*i*) VEGF content of culture medium of control and MDA-MB-231-β1 cells 1–3 days after plating (*n* = 6). Data are mean ± SEM; **p < 0.01; ***p < 0.001.

We next studied the structure and composition of the primary tumours. Both MDA-MB-231 and MDA-MB-231-β1 tumours were broadly similar, containing some invasion into surrounding fibroadipose tissue and skeletal muscle ( Fig. 2*f*). Although the *in vitro* invasiveness of MDA-MB-231-β1 cells was moderately higher than control MDA-MB-231 cells, blockade of α subunits with TTX inhibited invasion of both cell lines to a similar extent (*p* < 0.05, Fig. 2*g*). Thus, α-subunit-dependent invasion of MDA-MB-231 cells^20^,^21^ appears to be unaffected by β1 over-expression. The density of Ki67^+^ cycling cells was unchanged in MDA-MB-231-β1 tumours, compared to control tumours ( Figs. 3*a* and 3*b*). However, the density of apoptotic cells expressing activated caspase-3 was significantly reduced by 84% in MDA-MB-231-β1 tumours, compared to control tumours (*p* < 0.001; Figs. 3*c* and 3*d*). *In vitro*, staurosporine-induced apoptosis was significantly reduced in MDA-MB-231-β1 cells, compared to control MDA-MB-231 cells ( Figs. 3*e* and 3*f*), suggesting, together with the tumour data, that β1 over-expression enhances resistance to apoptosis. Finally, the density of vascular structures, revealed by labelling blood vessels with an antibody to the endothelial marker CD31, significantly increased by 1.5-fold in MDA-MB-231-β1 tumours, compared to control tumours, and VEGF secretion *in vitro* was significantly higher in MDA-MB-231-β1 cells than control MDA-MB-231 cells (*p* < 0.01; Figs. 3*g*−3*i*). In summary, these data suggest that β1 over-expression increased the growth of MDA-MB-231 tumours, not by altering the density of cycling cells in the population, but, instead by reducing apoptosis and enhancing angiogenesis.

### β1 potentiates metastasis to liver and lungs

**Figure 4:**
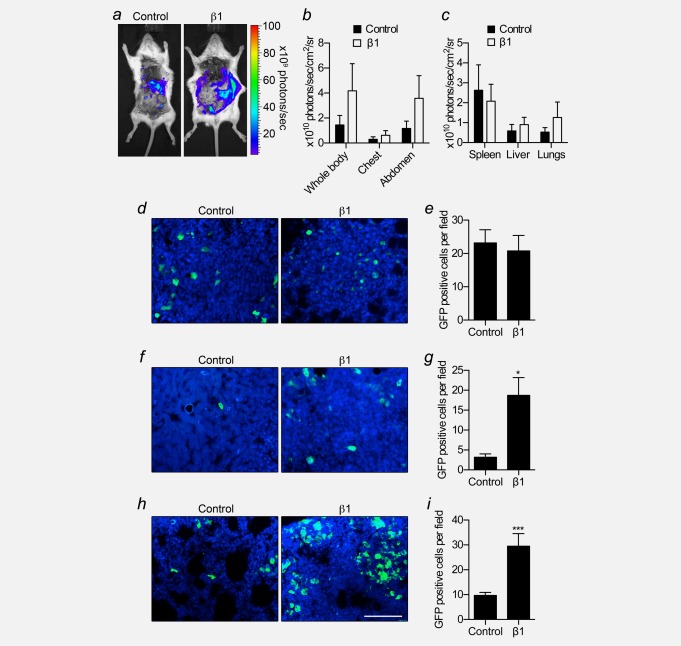
Effect of β1 over-expression on breast cancer metastasis *in vivo*. At 5 weeks following implantation, control and β1 tumours were removed *post mortem* prior to bioluminescent imaging. (*a*) Representative bioluminescent images of metastases from control and β1 tumours. (*b*) Bioluminescence measured from the indicated anatomical sites from control and β1 tumour-bearing mice (*n* ≥ 5). (*c*) Bioluminescence measured *ex vivo* from the spleen, liver, and lungs of control and β1 tumour-bearing mice (*n* ≥ 5). (*d*) Spleen sections from control and β1 tumour-bearing mice showing GFP signal (green) and DAPI (blue). (*e*) GFP-positive cells per field of view of spleen (*n* = 30). (*f*) Liver sections from control and β1 tumour-bearing mice showing GFP signal (green) and DAPI (blue). (*g*) GFP-positive cells per field of view of liver (*n* = 30). (*h*) Lung sections from control and β1 tumour-bearing mice showing GFP signal and DAPI (blue). (*i*) GFP-positive cells per field of view of lung (*n* = 30). Bars are mean + SEM; *p < 0.05; ***p < 0.001. Scale bar, 100 µm.

We monitored metastasis after 5 weeks by bioluminescent imaging following *post mortem* resection of primary tumours ( Fig. 4*a*). Although photon flux was slightly increased in mice bearing MDA-MB-231-β1 tumours compared those bearing MDA-MB-231 tumours, and in the liver and lungs *ex vivo*, this difference was not statistically significant ( Figs. 4*b* and 4*c*). To study metastasis to these organs in more detail at the cellular level, using a more sensitive method, we measured the density of GFP-expressing tumour cells within tissue sections. We detected GFP^+^ cells in sections both in isolation, and in multicellular foci ( Figs. 4*d*, 4*f*, and 4*h*). GFP was co-expressed in cells with HNA (Fig. S3*e*). HNA is present in human MDA-MB-231 cells, but absent in mouse cells, thus confirming that GFP expression was retained in the tumour cells once they had metastasized. In the spleen, the number of GFP^+^ cells per field of view was unchanged between groups ( Fig. 4*e*). However, the number of GFP^+^ cells per field of view was significantly increased, by 5.9- and 3.0-fold, respectively, in the liver and lungs of MDA-MB-231-β1 tumour-bearing mice, compared to control (*p* < 0.05 and 0.001, respectively; Figs. 4*g* and 4*i*). Thus, β1 over-expression promoted metastasis to the liver and lungs, but not the spleen.

### β1 promotes process outgrowth

**Figure 5:**
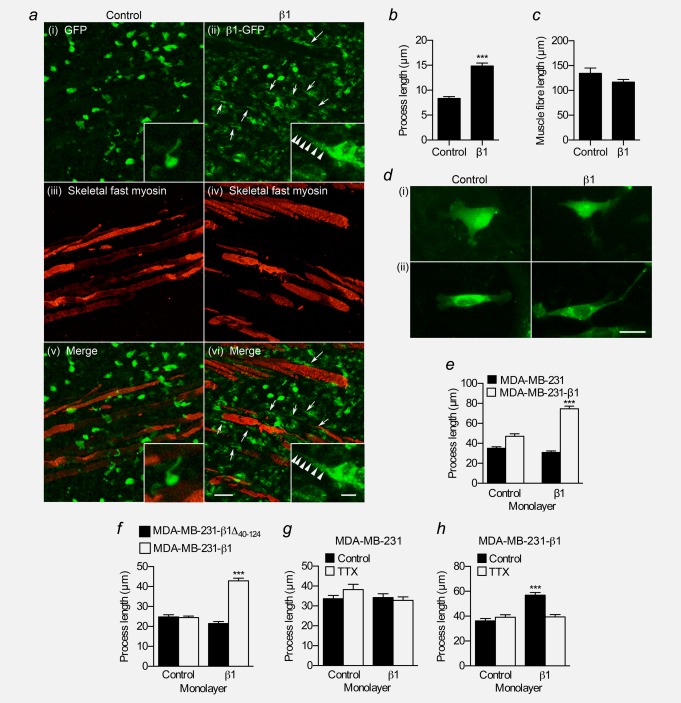
Effect of β1 on process outgrowth in breast cancer cells. (*a*) Regions of skeletal muscle infiltration in control (i,iii,v) and β1 (ii,iv,vi) tumour sections showing GFP signal (green) and skeletal fast myosin (red). Arrows indicate cells in β1 tumours that have more elongate processes. Scale bar, 50 µm. Insets, higher magnification images of tumour cells showing processes. Inset scale bar, 10 µm. (*b*) Process length (µm) of control MDA-MB-231 and MDA-MB-231-β1 cells in tumours (*n* ≥ 134 cells/each). (*c*) Length (µm) of muscle fibers in control and β1 tumours (*n* ≥ 79). (*d*) Images of MDA-MB-231 and MDA-MB-231-β1 cells grown on control or β1-expressing CHL fibroblast monolayers, and stained with anti-GFP antibody. Scale bar, 20 µm. (*e*) Process length (µm) of MDA-MB-231 and MDA-MB-231-β1 cells grown on control or β1-expressing CHL monolayers (*n* = 300). (*f*) Process length (µm) of MDA-MB-231-β1Δ_40–124_ and MDA-MB-231-β1 cells grown on control or β1-expressing CHL monolayers (*n* = 300). (*g*) Process length (µm) of MDA-MB-231 cells grown on control or β1-expressing CHL monolayers ± 30 µ*M* TTX (*n* ≥ 144). (*h*) Process length (µm) of MDA-MB-231-β1 cells grown on control or β1-expressing CHL monolayers ± 30 µM TTX (*n* = 150). Bars are mean + SEM; ***p < 0.001.

Enhancement of protrusions, *e.g*., pseudopodia, from cells is associated with increased motility in 3D cultures, invasion, and metastasis.^35^–^37^ Over-expression of β1 in MDA-MB-231 cells increases the length of processes protruding from the cell body *in vitro*.^24^ We therefore postulated that β1 might regulate cellular morphology in our tumour model. In the periphery of tumour sections, MDA-MB-231-β1 cells infiltrating surrounding skeletal muscle appeared more densely packed, and had a more elongate morphology than MDA-MB-231 cells ( Fig. 5*a*). In these sections, processes extending from MDA-MB-231-β1 cells were significantly longer than processes on MDA-MB-231 cells ( Fig. 5*b*). The length of muscle fibers was unchanged between tumour types ( Fig. 5*c*).

In the nervous system, β1 regulates neuronal morphology and neurite outgrowth *via trans*-homophilic adhesion.^15^ We therefore set out to test the hypothesis that this neuronal function of β1 is recapitulated when it is expressed in BCa cells, enhancing process outgrowth. We examined the morphology of BCa cells plated on monolayers of control and β1-expressing CHL fibroblasts. CHL cells were chosen because they do not express endogenous β subunits.^14^ When plated on CHL cells, MDA-MB-231 cells produced thin processes with foci at the tips, morphologically similar to neurites with growth cones (Fig. 5*d*).^15^ MDA-MB-231 cells did not show any increase in process length when grown on β1-expressing monolayers ( Fig. 5*e*). However, MDA-MB-231-β1 cells did respond, such that β1-expressing monolayers increased process length by 1.6-fold (*p* < 0.001; Fig. 5*e*). A similar result was observed for MCF-7 cells, which express endogenous β1 (Supporting Information Fig. S4*a* and S4*b*). There was no increase in process length of MDA-MB-231 cells over-expressing the Ig domain-deficient mutant β1Δ_40–124_ when grown on β1-expressing monolayers ( Fig. 5*f*), suggesting that the adhesion domain is required for β1-mediated process outgrowth. In cerebellar granule neurons, β1-mediated neurite outgrowth requires the presence of Na_v_1.6 and is inhibited by the VGSC pore-blocking toxin TTX.^16^ We found that TTX (30 µ*M*) inhibited β1-mediated process outgrowth in MDA-MB-231-β1 cells (*p* < 0.001; Fig. 5*h*). However, it had no effect on process outgrowth in native MDA-MB-231 cells, which do not respond to β1-expressing fibroblasts (Fig. 5*g*). Together, these data suggest that β1 enhances process outgrowth on BCa cells *via trans*-homophilic adhesion between β1 expressed on the BCa cell, and β1 expressed on the adjacent fibroblast, similar to its function in neurons.^14^

### β1-mediated process outgrowth requires fyn kinase

**Figure 6:**
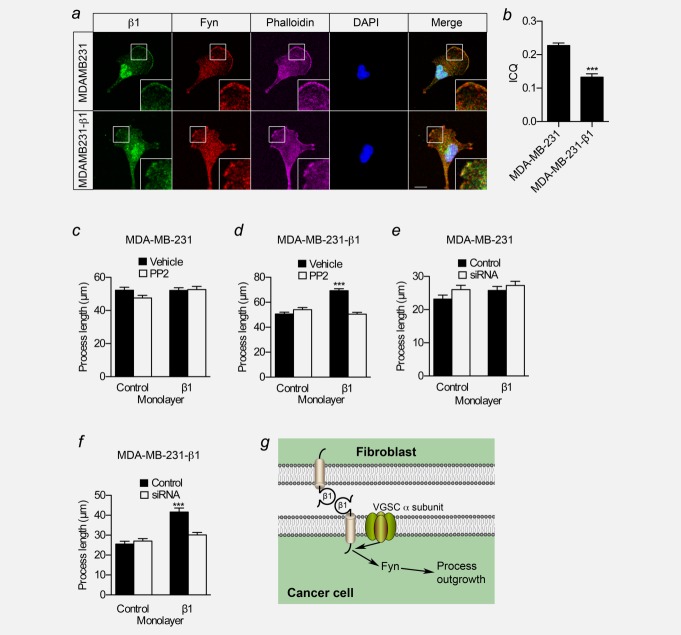
A mechanism for β1-mediated process outgrowth and migration in BCa cells. (*a*) Images of MDA-MB-231 and MDA-MB-231-β1 cells. Green: anti-β1 for parental MDA-MB-231 (with Alexa-488-conjugated secondary antibody) and GFP signal for MDA-MB-231-β1; red: anti-fyn; magenta: phalloidin to label the actin cytoskeleton; blue: DAPI to label nucleus. White boxes indicate locations of inset zoomed images. Phalloidin is omitted from merged image for clarity. Scale bar, 10 µm. (*b*) Intensity correlation-quotients (ICQ) for β1 and fyn in MDA-MB-231 and MDA-MB-231-β1 cells (*n* = 20/each). (*c*) Process length (µm) of MDA-MB-231 cells grown on control or β1-expressing CHL monolayers ± 5 µM PP2 (*n* ≥ 147). (*d*) Process length (µm) of MDA-MB-231-β1 cells grown on control or β1-expressing CHL monolayers ± 5 µ*M* PP2 (*n* ≥ 223). (*e*) Process length (µm) of MDA-MB-231 cells grown on control or β1-expressing CHL monolayers ± fyn siRNA (*n* = 150). (*f*) Process length (µm) of MDA-MB-231-β1 cells grown on control or β1-expressing CHL monolayers ± fyn siRNA (*n* = 150). (*g*) A model of possible signalling mechanism underlying β1-mediated process outgrowth in BCa cells. β1 from an adjacent fibroblast or cancer cell interacts in *trans* with β1 on the BCa cell, initiating a signaling cascade *via* fyn kinase, leading to process outgrowth.^15^ Na^+^ conductance through the pore-forming α subunit is also required.^16^ Figure was produced using Science Slides software. Bars are mean + SEM; ***p < 0.001.

In neurons, β1 increases neurite length *via* fyn kinase.^15^ CAM-mediated activation of fyn kinase is proposed to initiate the fyn-focal adhesion kinase (FAK) pathway, activating extracellular signal-regulated kinase 1/2, leading to neurite outgrowth.^38^ Fyn is upregulated in a number of cancers, where it contributes to an invasive phenotype.^39^ We found that fyn, like β1, was expressed in MCF-10A cells and BCa cell lines (Fig. 1*g* and 1*h*, Supporting Information Fig. S2*f*). Given that fyn is required for β1-mediated neurite outgrowth, and fyn and β1 are coexpressed in brain membranes,^15^ we hypothesized that fyn and β1 may colocalize in BCa cells. In MDA-MB-231 and MDA-MB-231-β1 cells, β1 was expressed throughout the cytoplasm, on perinuclear internal membranes and lamellipodia ( Fig. 6*a*), consistent with previous observations, although the expression of β1 was clearly lower in the former.^23^ Importantly, fyn showed a broadly similar distribution to β1, with expression strongest on F-actin-rich lamellipodia. Intensity correlation analysis gave ICQ values >0, indicating that the signals for β1 and fyn varied together^30^ ( Fig. 6*b*). This result is consistent with β1 colocalizing with fyn.

We next investigated whether fyn activity is involved in β1-mediated process outgrowth in BCa cells. Inclusion of the src family kinase inhibitor PP2 (5 µ*M*) in the assay inhibited β1-mediated process outgrowth in MDA-MB-231-β1 cells (*p* < 0.001; Fig. 6*d*). However, it had no effect on process outgrowth in control MDA-MB-231 cells, which do not respond to β1-expressing fibroblasts ( Fig. 6*c*). Importantly, 5 µ*M* PP2 had no effect on cellular viability or proliferation (Supporting Information Figs. S5*a* and S5*b*). To establish whether fyn is specifically required for β1-mediated process outgrowth in BCa cells over other members of the src family that are also inhibited by PP2, we next transiently down-regulated the expression of fyn in BCa cells using siRNA, prior to plating on fibroblast monolayers (Supporting Information Fig. S5*d*). Down-regulation of fyn with siRNA abrogated β1-mediated process outgrowth in MDA-MB-231-β1 cells (*p* < 0.001; Fig. 6*f*). However, there was no effect on baseline process outgrowth in control MDA-MB-231 cells ( Fig. 6*e*). PP2 and fyn siRNA also inhibited β1-mediated process outgrowth in MCF-7 cells (Supporting Information Figs. S4*c* and S4*d*). In summary, blocking fyn expression/activity with PP2 or siRNA inhibited β1-mediated process outgrowth in BCa cells. These data suggest that fyn is a critical signalling intermediary in the mechanism underlying β1-mediated process outgrowth in BCa cells (Fig. 6*g*), as it is in neurons.^15^

## Discussion

VGSCs are expressed in cells from a number of different cancers, where they are proposed to play a role in potentiating metastasis.^18^ VGSCs are unique among ion channels in that their “auxiliary” β subunits not only modulate channel activity, but are also CAMs.^5^ Expression of β subunits has been reported in breast, bone, cervical, colorectal, lung, and prostate cancer cell lines, and β1 is the dominant isoform in breast, cervical, lung and prostate cancer cell lines (reviewed in Ref.^19^). However, *in vivo* evidence for β subunit expression in cancer is limited. We found that β1 was up-regulated at mRNA and protein level in BCa specimens compared with non-cancer tissue. Similarly, β1 was expressed across a panel of BCa cell lines, although the relative mRNA and protein levels differed. In addition, in tumour specimens, the positive relationship between *SCN1B* mRNA and ER status was not reflected at the protein level. Discrepancy between mRNA and protein levels has been reported previously for other VGSCs in other tissues.^24^,^40^,^41^ Therefore, the relationship between *SCN1B* mRNA and β1 protein levels may be subject to complex regulation, highlighting the critical importance of studying biomarker expression at both mRNA and protein levels. We did not observe a relationship between β1 expression and outcome in patient tumour specimens. This may be due to the relatively small size of the dataset, and it would be worthwhile in the future to validate the data presented here against larger cohorts. In conclusion, our data show that *SCN1B*/β1 is up-regulated at the mRNA and protein level in BCa. We propose that β1 warrants further study as a potential biomarker for BCa.

β1 over-expression increased tumour growth *in vivo*. Interestingly, this contrasts with the observation that over-expression of β1 slightly reduces proliferation *in vitro,*^24^ suggesting that the tumour microenvironment might be critical to the *in vivo* tumour-promoting function of β1. In support of this, apoptosis was reduced in MDA-MB-231-β1 tumours, which may account for their increased size. Cell adhesion can promote apoptosis suppression in cancer cells via FAK activation^42^ and further work is required to establish whether or not β1-dependent adhesion promotes tumour cell survival. There was increased density of vascular structures in the β1 over-expressing tumours, and VEGF secretion was increased in MDA-MB-231-β1 cells *in vitro*, suggesting that β1 may enhance angiogenesis. Several classes of CAMs are known to promote angiogenesis, including integrins, cadherins, and immunoglobulin superfamily CAMs,^43^ raising the possibility that β1 may contribute to promoting blood vessel development through heterophilic adhesion. Interestingly, over-expression of β2 in prostate cancer cells has the reverse effect, reducing tumour growth.^44^ Despite its structural similarity to β1, β2 appears to play a different role in the CNS, and is not essential for postnatal development.^5^ Thus, as in the CNS, different β subunits may perform distinct functions in different cancer microenvironments.

β1 is a multifunctional molecule that plays a critical role during CNS development.^5^ Although β1 is essential for regulating excitability through fine-tuning VGSC gating and kinetics,^16^ its function as a CAM is required for neurite outgrowth, migration, fasciculation and synaptic connectivity.^15^–^17^ In fact, β1 may function as a CAM, independent of channel activity under certain conditions.^5^ Other CAMs that regulate neuronal migration and pathfinding have been reported in tumours, where they play a pathological role and associate with poor prognosis.^45^ It is therefore not unreasonable to expect that β1 may do the same. We showed previously that β1 enhances cell-cell adhesion and cell-substrate adhesion in BCa cells *in vitro*.^24^ In the present study, we found that β1 over-expression in MDA-MB-231 cells caused a more elongate cellular morphology within tumours, and enhanced process outgrowth *in vitro via trans-*homophilic adhesion. β1-mediated process outgrowth did not occur in control MDA-MB-231 cells, which express a low level of endogenous β1. The latter result suggests that β1 expression on the tumour cell may need to be above a threshold in order to induce process outgrowth and enhance tumour growth and metastasis. We found that, as in neurons,^15^ β1-mediated process outgrowth in BCa cells required fyn kinase. β1-mediated neurite outgrowth in neurons is also activity-dependent.^16^ Interestingly, Na^+^ current promotes src family kinase activity and pro-invasive morphology in MDA-MB-231 cells,^46^ which fits with other data showing that α subunits potentiate the invasiveness of BCa cells.^20^–^22^,^46^,^47^ We found that TTX inhibited β1-mediated process outgrowth in MDA-MB-231-β1 cells, suggesting that, as in neurons,^16^ α subunit function is required (Fig. 6*g*). Thus, Na_v_1.5 and β1 may both promote mesenchymal-like elongate morphology in BCa cells, *via* a combination of Na^+^ current and adhesion.

Our data suggest that β1 can enhance tumour growth and metastasis *via* a *trans*-homophilic adhesion mechanism that enhances process outgrowth on metastatic tumour cells, enabling their dissemination from the primary tumour and into surrounding tissues. This would fit with the observation that outgrowth of processes, *e.g*., pseudopodia, from cancer cells increases motility, invasion, and metastasis.^35^–^37^ Thus, β1 may be involved in collective cell migration and invasion during tumour spreading,^48^,^49^ similar to its role in pathfinding and fasciculation during CNS development.^15^,^17^ However, we do not yet know whether β1 interactions in *trans* occur between adjacent tumour cells, or between tumour cells and stromal cells, or both. Further complexity is added by the possibility that β1 may interact heterophilically with other CAMs,^9^–^12^ and/or extracellular matrix proteins^13^ present in the tumour microenvironment, dependent on cell types/status within the tumour.

Our data support the hypothesis that *SCN1B*/β1 recapitulates its neurodevelopmental role to promote breast tumour growth and metastasis. This fits with a growing body of evidence implicating VGSCs as mediators of an invasive/metastatic phenotype.^19^ Up-regulation of genes, *e.g*. *SCN1B*, required for normal migration and invasion processes during development, may represent a critical event in the progression towards metastasis.^50^ We therefore propose that (*i*) β1 may represent a novel biomarker during disease development, and (*ii*) targeting β1-mediated adhesion interactions may have potential as novel anti-cancer therapy.

## Abbreviations

BCa: breast cancer
CAM: cell adhesion molecule
CHL: Chinese hamster lung
CNS: central nervous system
DMEM: Dulbecco's modified eagle medium
ER: estrogen receptor α
FAK: focal adhesion kinase
GFP: green fluorescent protein
HNA: human nuclear antigen
ICA: intensity correlation analysis
ICC: immunocytochemistry
ICQ: intensity correlation quotient
IHC: immunohistochemistry
VGSC: voltage-gated Na^+^ channel

## Supporting Information

Supplementary Information Figure 1. *SCN1B* mRNA expression in breast cancer. (A) *SCN1B* expression profile in the Lu Breast dataset, segregated by ER status (n=129). (B) *SCN1B* expression vs. *ESR1* expression in the Lu Breast dataset. (C) *SCN1B* expression in the context of genomic neighbours. Genes are sorted by their genomic distance to *SCN1B* (indicated underneath matrix). Colours indicate mean fold change in expression (from 0.65-1.8) in ER+ cases relative to ER^-^ cases across five Oncomine datasets. (D) β1 and (E) β1B mRNA expression across a panel of BCa cell lines (qPCR relative to GAPDH, calibrator=SKBR3; n=3). (F) Expression of β1, β1B, and GREB1 (relative to GAPDH) in MCF-7 cells treated with vehicle, or 10nM estradiol and/or 1μM fulvestrant (n=3). Estradiol significantly increased mRNA level (reversed by fulvestrant) of the positive control, *GREB1*, an estrogen-regulated gene. Box plot dots, maximum and minimum values; whiskers, 90^th^ and 10^th^ percentile values; and horizontal lines, 75^th^, 50^th^, and 25^th^ percentile values. **P<0.01; ***P<0.001.

Supplementary Information Figure 2. β1, fyn, CD44 and E-cadherin protein levels in MDA-MB-231 cells. (A) Western blot of β1 in control MDA-MB-231 cells (expressing eGFP) and MDA-MB-231-β1 cells (overexpressing β1-eGFP C-terminal fusion, 67 kDa). (B) Protein levels of β1 and β1-GFP, relative to ?-tubulin (n=3 repeats). Bars are mean + SEM. ***P<0.001. (C) Images of control MDA-MB-231 cells and MDA-MB-231-β1 cells labelled with anti-CD44 and anti-E-cadherin antibodies (red), and DAPI to label the nucleus. Positive control for E-cadherin labelling, MCF-7 cells. Scale bar, 50μm. (D) Western blot of CD44 in control MDA-MB-231 cells and MDA-MB-231-β1 cells. (E) Western blot of E-cadherin in control MDA-MB-231 cells and MDA-MB-231-β1 cells. Positive control=MCF-7 cells. (F) Western blot of fyn kinase in control MDA-MB-231 cells and MDA-MB-231-β1 cells.

Supplementary Information Figure 3. Luciferase and GFP expression in MDA-MB-231 cells. (A) Representative bioluminescent image of luciferase activity in cultured control MDA-MB-231 cells and MDA-MB-231-β1 cells (1x105 cells/well), 10 min following addition of D-luciferin (1 mg/ml). (B) Bioluminescence measured from cultured cells (n=6 wells/group). (C) Images showing eGFP expression (green) in control MDA-MB-231 cells and MDA-MB-231-β1 cells counterstained with DAPI (blue). Scale bar, 50μm. (D) Quantification of eGFP signal intensity per cell (n=50). Individual cells were delineated using the freeform line tool in ImageJ in order to obtain a pixel intensity score per cell. (E) Metastasis in lung section from a β1 tumour-bearing mouse showing GFP expression (green) in tumour cells overlaying with human nuclear antigen (HNA) expression (red), counterstained with DAPI (blue). White dashed lines delineate extent of metastasis. Scale bar, 100μm. Data are mean + SEM.

Supplementary Information Figure 4. β1-mediated process outgrowth in MCF-7 cells. (A) Typical images of MCF-7 cells grown on control or β1-expressing CHL fibroblast monolayers. MCF-7 cells were visualised by staining with anti-cytokeratin 18 antibody (red). Scale bar, 20μm. (B) Process length (μm) of MCF-7 cells grown on control or β1-expressing CHL monolayers (n=300). (C) Process length (μm) of MCF-7 cells grown on control or β1-expressing CHL monolayers ± 5μM PP2 (n=180). (D) Process length (μm) of MCF-7 cells grown on control or β1-expressing CHL monolayers ± fyn siRNA (n=150). Bars are mean + SEM; ***P<0.001.

Supplementary Information Figure 5. Targeting fyn expression/activity. (A) Viability (%) of MCF-7 and MDA-MB-231 cells following treatment with PP2 (0.5, 1, 5, 10, 30μM) or vehicle for 24h, normalised to control (n=60). (B) Proliferation of MCF-7 and MDA-MB-231 cells following treatment with PP2 (0.5, 1, 5, 10, 30μM) or vehicle for 24h, normalised to control (n=9). (C) GAPDH mRNA expression in MCF-7 and MDA-MB-231 cells following transfection with non-targeting control siRNA (“Control”) or siRNA targeting GAPDH (“siRNA”). QPCR relative to β-actin (n=3). In both cell lines, down-regulation of GAPDH expression with siRNA indicated transfection efficiency ≥90%. (D) Fyn mRNA expression in MCF-7, MDA-MB-231 and MDA-MB-231-β1 cells following transfection with non-targeting control siRNA (“Control”) or siRNA targeting fyn kinase (“siRNA”). QPCR relative to GAPDH (n=3 repeat transfections). Data are mean ± SEM; ***P<0.001.

Supplementary Information Table 1. Primer sequences.

Supplementary Information Table 2. Correlation between SCN1B expression and histoclinical characteristics

Supplementary Information Table 3. Patient histoclinical characteristics and β1 expression.
