# Supplementary figures and images for: The sodium channel β1 subunit mediates outgrowth of neurite-like processes on breast cancer cells and promotes tumour growth and metastasis
Source: Int J Cancer. 2014 Apr 12;135(10):2338–51. doi: 10.1002/ijc.28890 (PMC4200311; doi:10.1002/ijc.28890)

A

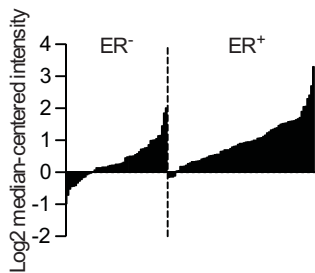

B

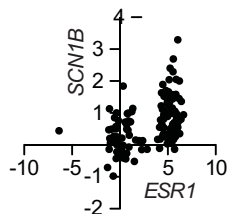

C

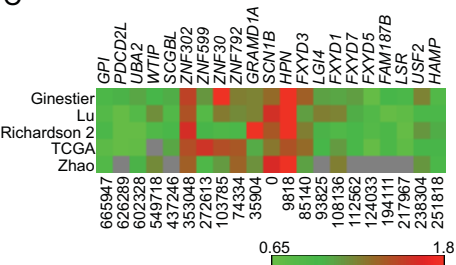

D

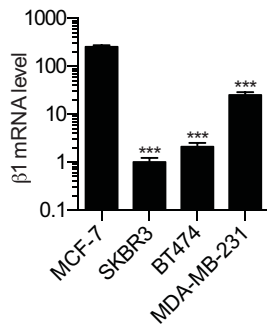

E

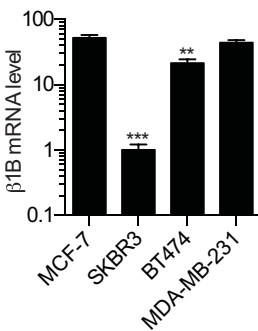

F

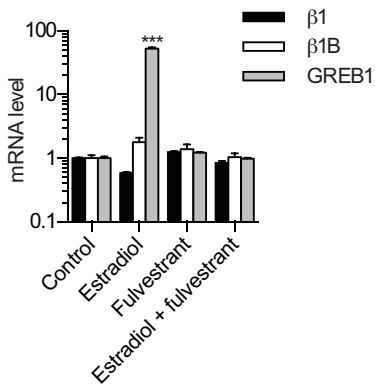

Supplement: Supplementary file 1 — Supplementary Information Figure 1. SCN1B mRNA expression in breast cancer. (A) SCN1B expression profile in the Lu Breast dataset, segregated by ER status (n=129). (B) SCN1B expression vs. ESR1 expression in the Lu Breast dataset. (C) SCN1B expression in the context of genomic neighbours. Genes are sorted by their genomic distance to SCN1B (indicated underneath matrix). Colours indicate mean fold change in expression (from 0.65-1.8) in ER+ cases relative to ER- cases across five Oncomine datasets. (D) β1 and (E) β1B mRNA expression across a panel of BCa cell lines (qPCR relative to GAPDH, calibrator=SKBR3; n=3). (F) Expression of β1, β1B, and GREB1 (relative to GAPDH) in MCF-7 cells treated with vehicle, or 10nM estradiol and/or 1μM fulvestrant (n=3). Estradiol significantly increased mRNA level (reversed by fulvestrant) of the positive control, GREB1, an estrogen-regulated gene. Box plot dots, maximum and minimum values; whiskers, 90th and 10th percentile values; and horizontal lines, 75th, 50th, and 25th percentile values. **P<0.01; ***P<0.001. [file ijc0135-2338-SD1.pdf]

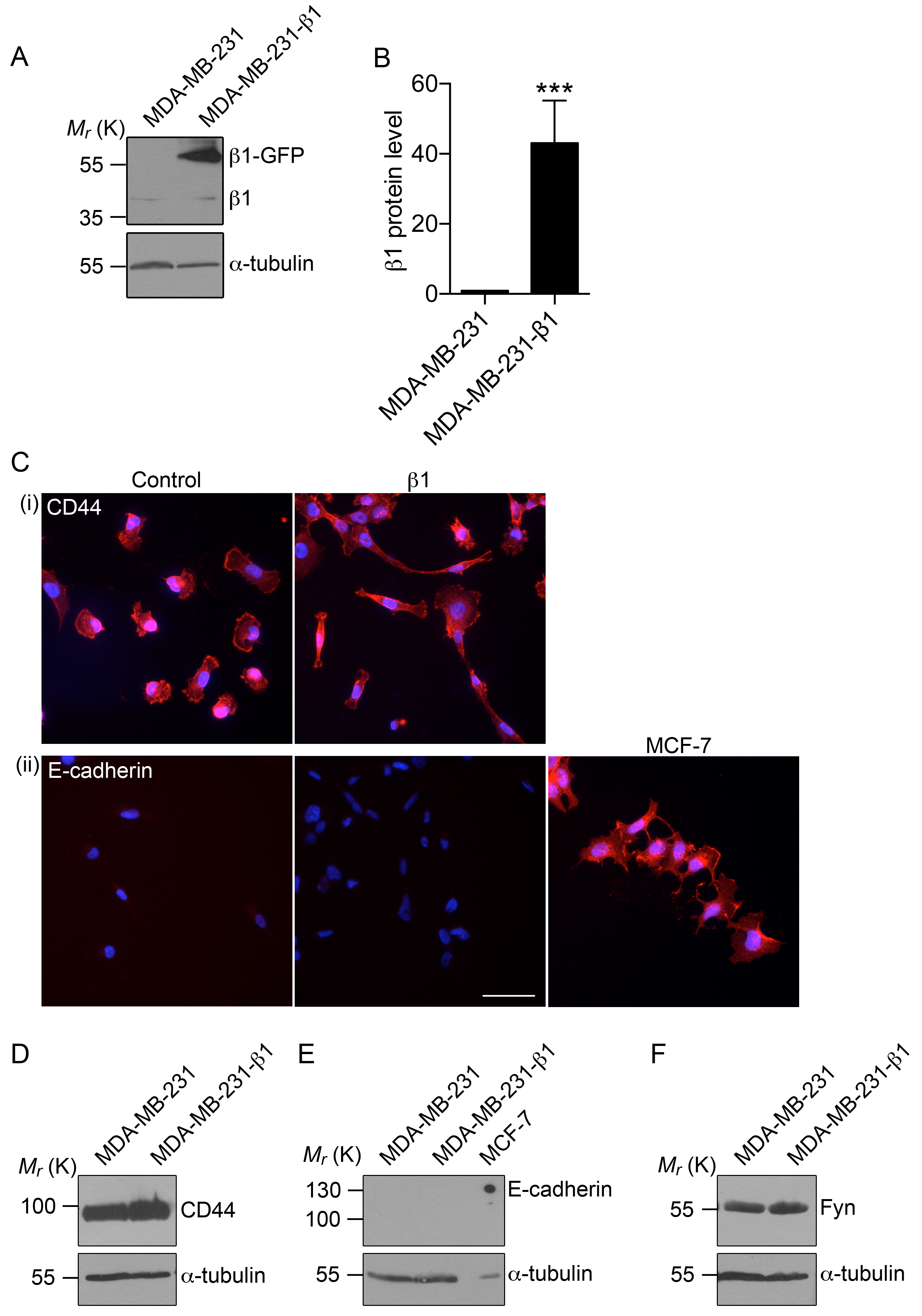

Supplement: Supplementary file 3 — Supplementary Information Figure 2. β1, fyn, CD44 and E-cadherin protein levels in MDA-MB-231 cells. (A) Western blot of β1 in control MDA-MB-231 cells (expressing eGFP) and MDA-MB-231-β1 cells (overexpressing β1-eGFP C-terminal fusion, 67 kDa). (B) Protein levels of β1 and β1-GFP, relative to ?-tubulin (n=3 repeats). Bars are mean + SEM. ***P<0.001. (C) Images of control MDA-MB-231 cells and MDA-MB-231-β1 cells labelled with anti-CD44 and anti-E-cadherin antibodies (red), and DAPI to label the nucleus. Positive control for E-cadherin labelling, MCF-7 cells. Scale bar, 50μm. (D) Western blot of CD44 in control MDA-MB-231 cells and MDA-MB-231-β1 cells. (E) Western blot of E-cadherin in control MDA-MB-231 cells and MDA-MB-231-β1 cells. Positive control=MCF-7 cells. (F) Western blot of fyn kinase in control MDA-MB-231 cells and MDA-MB-231-β1 cells. [file ijc0135-2338-SD2.tif]

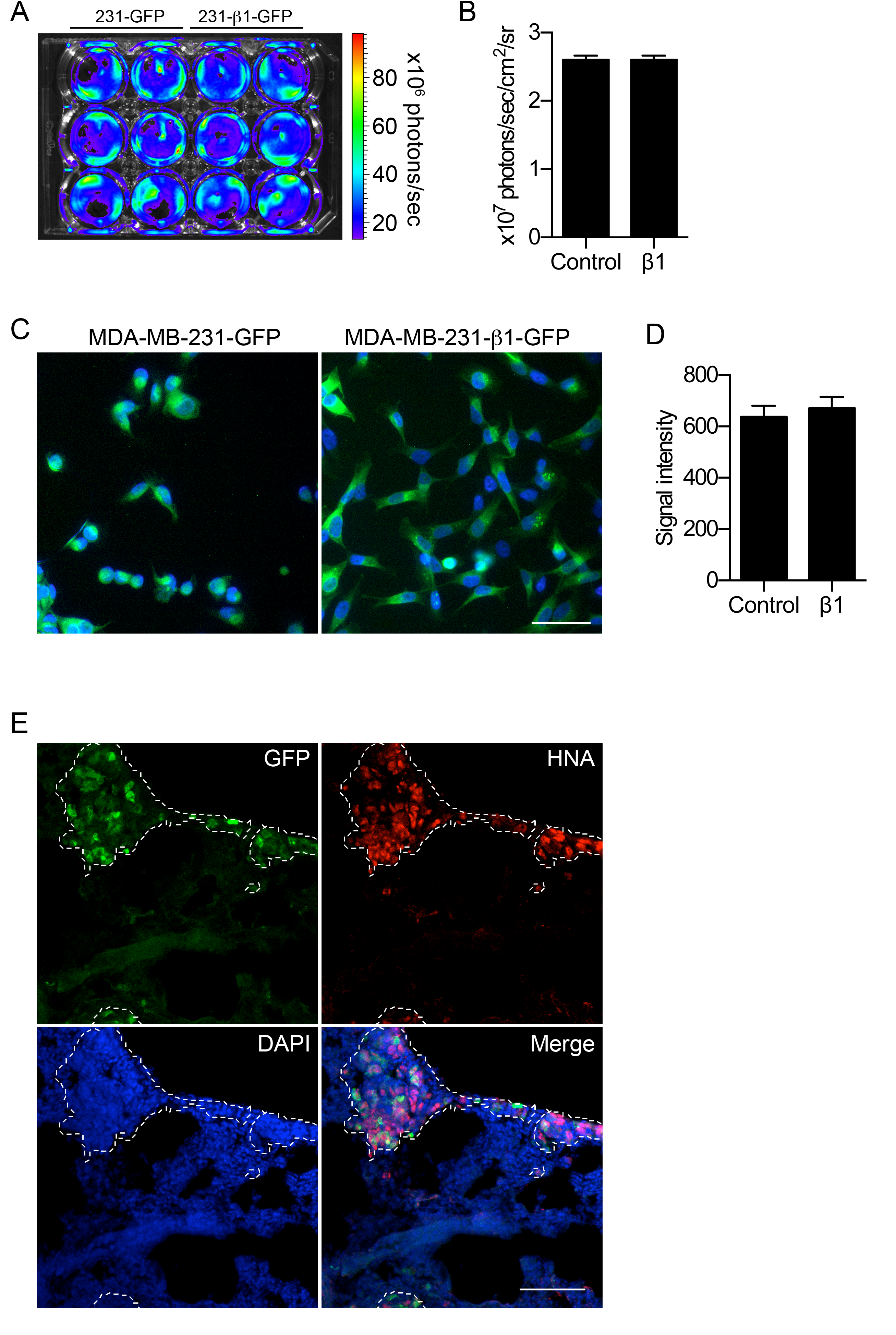

Supplement: Supplementary file 5 — Supplementary Information Figure 3. Luciferase and GFP expression in MDA-MB-231 cells. (A) Representative bioluminescent image of luciferase activity in cultured control MDA-MB-231 cells and MDA-MB-231-β1 cells (1x10^5^ cells/well), 10 min following addition of D-luciferin (1 mg/ml). (B) Bioluminescence measured from cultured cells (n=6 wells/group). (C) Images showing eGFP expression (green) in control MDA-MB-231 cells and MDA-MB-231-β1 cells counterstained with DAPI (blue). Scale bar, 50μm. (D) Quantification of eGFP signal intensity per cell (n=50). Individual cells were delineated using the freeform line tool in ImageJ in order to obtain a pixel intensity score per cell. (E) Metastasis in lung section from a β1 tumour-bearing mouse showing GFP expression (green) in tumour cells overlaying with human nuclear antigen (HNA) expression (red), counterstained with DAPI (blue). White dashed lines delineate extent of metastasis. Scale bar, 100μm. Data are mean + SEM. [file ijc0135-2338-SD3.tif]

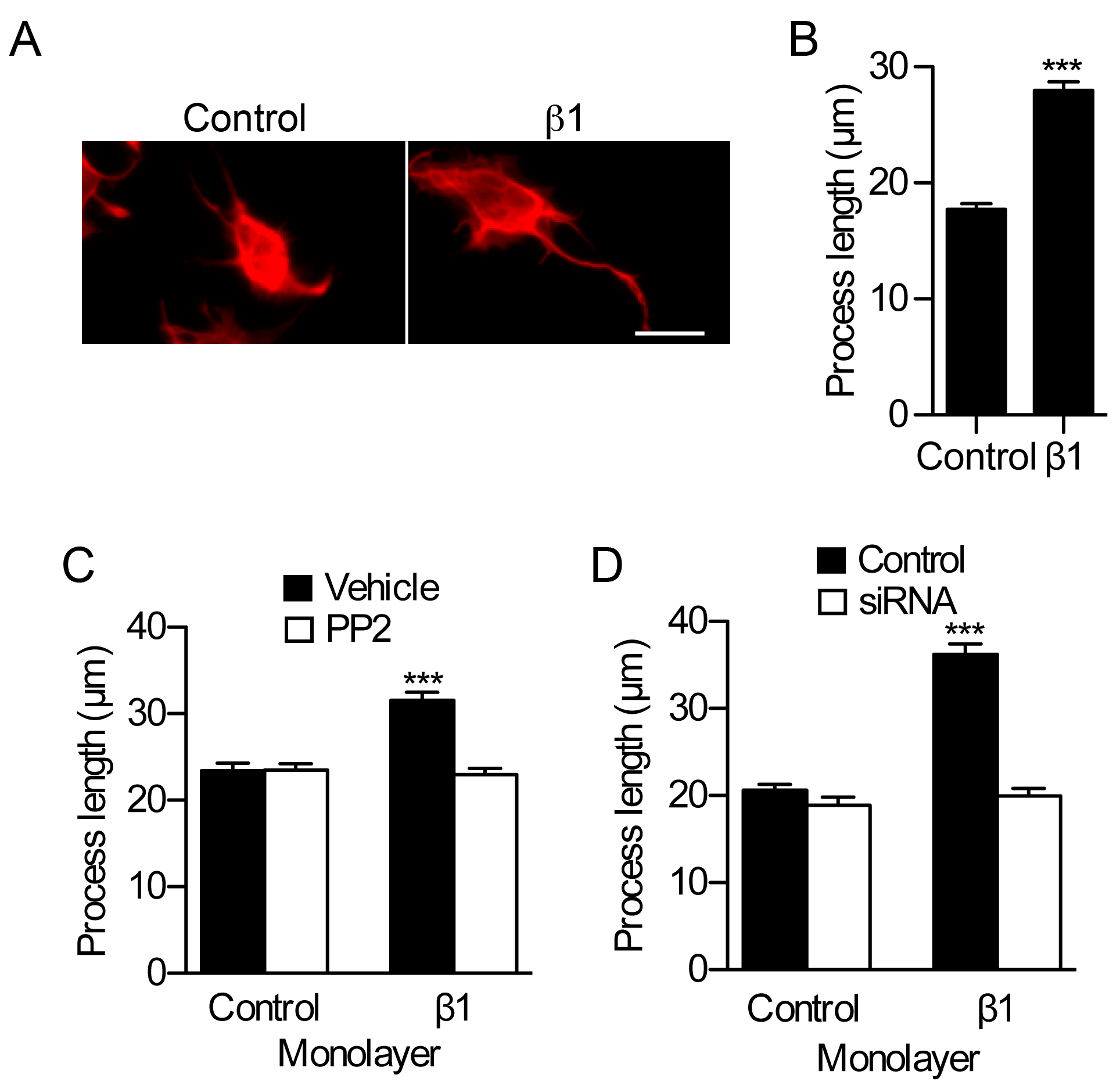

Supplement: Supplementary file 7 — Supplementary Information Figure 4. β1-mediated process outgrowth in MCF-7 cells. (A) Typical images of MCF-7 cells grown on control or β1-expressing CHL fibroblast monolayers. MCF-7 cells were visualised by staining with anti-cytokeratin 18 antibody (red). Scale bar, 20μm. (B) Process length (μm) of MCF-7 cells grown on control or β1-expressing CHL monolayers (n=300). (C) Process length (μm) of MCF-7 cells grown on control or β1-expressing CHL monolayers ± 5μM PP2 (n=180). (D) Process length (μm) of MCF-7 cells grown on control or β1-expressing CHL monolayers ± fyn siRNA (n=150). Bars are mean + SEM; ***P<0.001. [file ijc0135-2338-SD4.tif]

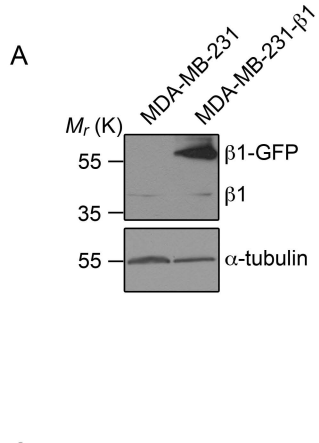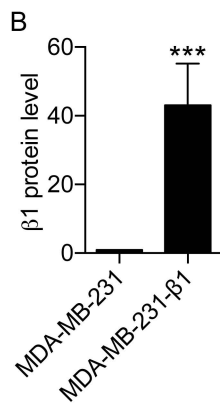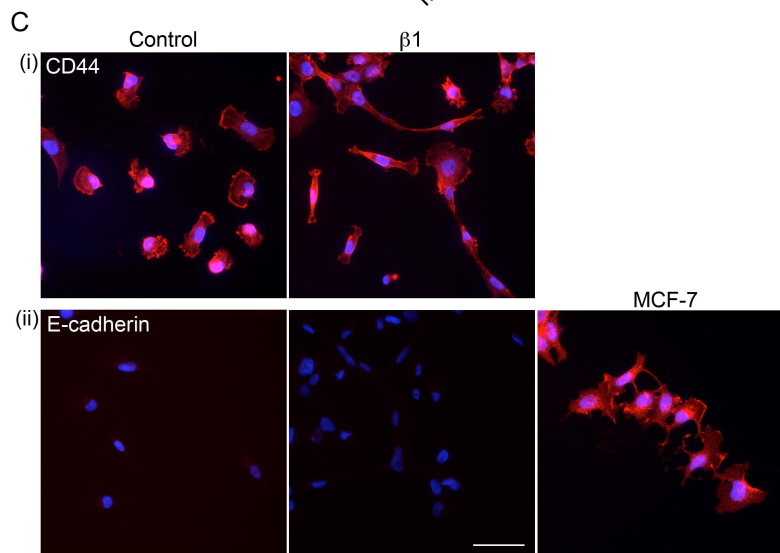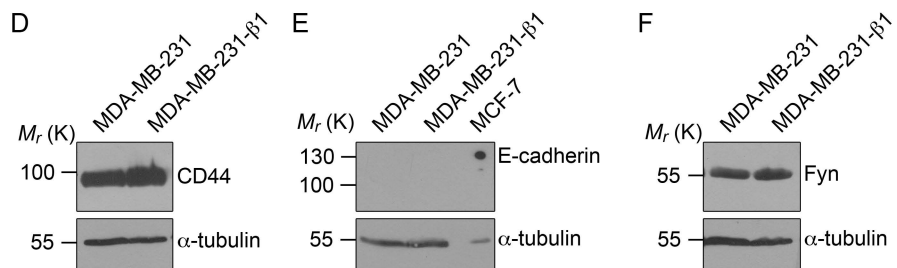

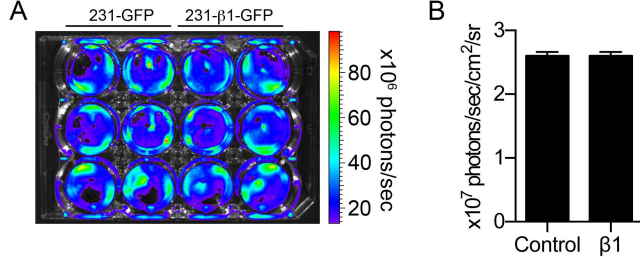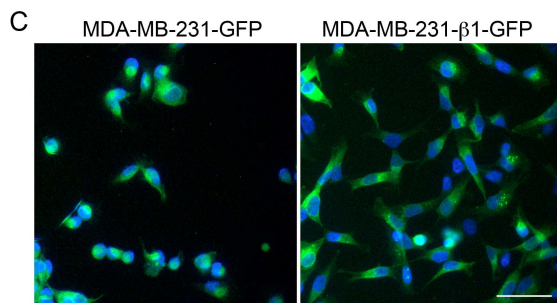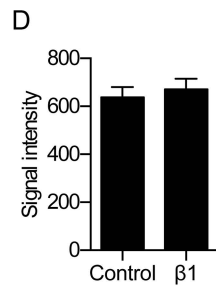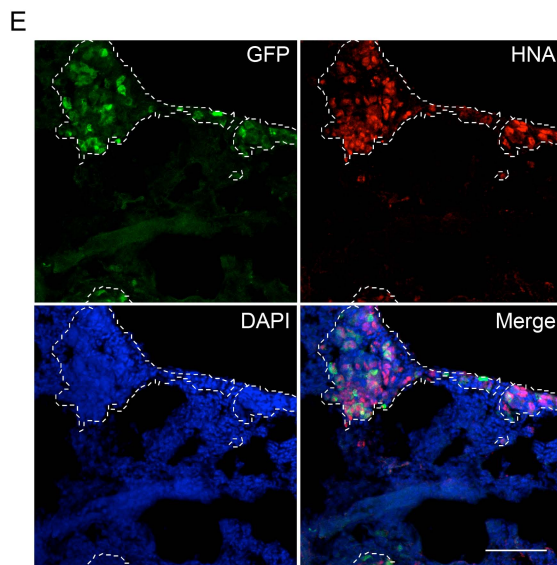

A

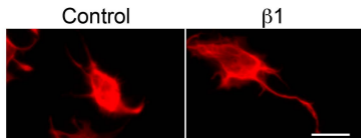

B

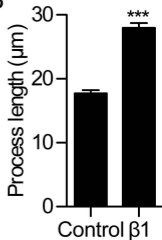

C

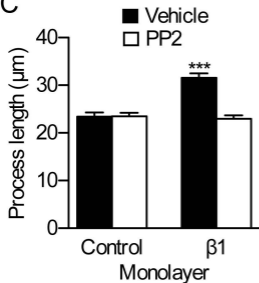

D

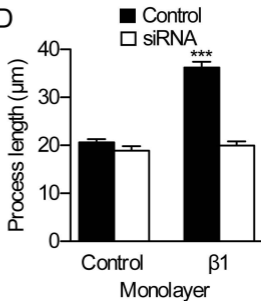

A

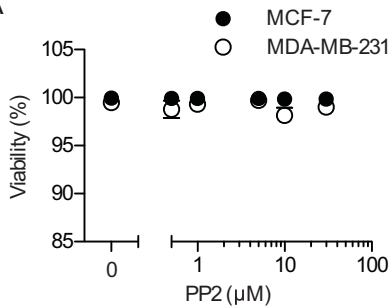

B

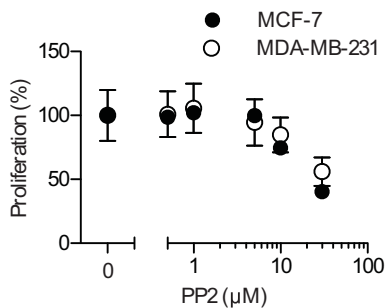

C

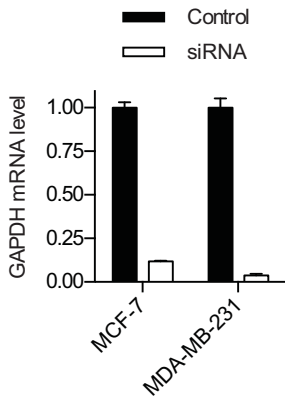

D

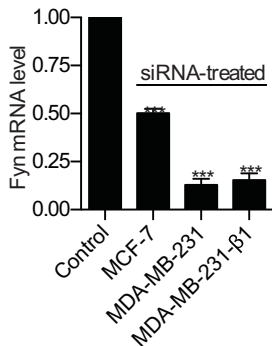

Supplement: Supplementary file 14 — Supplementary Information [file ijc0135-2338-SD9.pdf]
