# Supplementary material for: The sodium channel β1 subunit mediates outgrowth of neurite-like processes on breast cancer cells and promotes tumour growth and metastasis
Source: Int J Cancer. 2014 Apr 12;135(10):2338–51. doi: 10.1002/ijc.28890 (PMC4200311; doi:10.1002/ijc.28890)
Supplement: Supplementary file 11 — Supplementary Information Table 1. Primer sequences. [file ijc0135-2338-SD6.pdf]

Table S1. Primer sequences.

Gene	Forward primer 5'-3'	Reverse primer 5'-3'	Reference
ACTB	AGCCTCGCCTTTGCCGA	CTGGTGCCTGGGGCG	[1]
FYN	GCAGGATGCTGATCTAAACG	TCCTTACATTGCACACAGCC	-
GAPDH	AAGGTGAAGGTCGGAGTCAAC	CCAGAGTTAAAAGCAGCCCTG	[2]
GREB1	CAAAGAATAACCTGTTGGCCCTGC	GACATGCCTGCGCTCTCATACTTA	[3]
SCN1B - β1 splice variant	GTCGTCAAGAAGATCCACATTGAGGT	TTCGGCCACCTGGACGCCCGTGCAG	[4]
SCN1B - β1B splice variant	GTCGTCAAGAAGATCCACATTGAGGT	AACCACACCCCGAGAAACACATCGGA	[4]

References

1 Kreuzer, K. A. *et al.* Highly sensitive and specific fluorescence reverse transcription-PCR assay for the pseudogene-free detection of beta-actin transcripts as quantitative reference. *Clin Chem* **45**, 297-300 (1999).

2 Swift, S. L., Burns, J. E. & Maitland, N. J. Altered expression of neurotensin receptors is associated with the. *Cancer Res* **70**, 347-356, doi:10.1158/0008-5472.can-09-1252 (2010).

3 Rae, J. M. *et al.* GREB 1 is a critical regulator of hormone dependent breast cancer growth. *Breast Cancer Res Treat* **92**, 141-149, doi:10.1007/s10549-005-1483-4 (2005).

4 Patino, G. A. *et al.* Voltage-gated Na+ channel β1B: a secreted cell adhesion molecule involved in human epilepsy. *J Neurosci* **31**, 14577-14591 (2011).
