# Supplementary material for: The sodium channel β1 subunit mediates outgrowth of neurite-like processes on breast cancer cells and promotes tumour growth and metastasis
Source: Int J Cancer. 2014 Apr 12;135(10):2338–51. doi: 10.1002/ijc.28890 (PMC4200311; doi:10.1002/ijc.28890)
Supplement: Supplementary file 12 — Supplementary Information Table 2. Correlation between SCN1B expression and histoclinical characteristics [file ijc0135-2338-SD7.pdf]

Table S2. Correlation between *SCN1B* expression and histoclinical characteristics

Oncomine breast dataset	*SCN1B* level	Age		Pathological tumour size			Grade			Recurrence within 5 years		ER		PR		HER2	
		≤50	>50	T1	T2	T3	1	2	3	-	+	-	+	-	+	-	+
Bittner	Low	52	115	44	84	13	12	56	70			37	69	53	53	69	29
	High	46	122	40	75	25	18	51	71			41	85	55	69	83	31
	P	0.47		0.11			0.49					0.78		0.43		0.76	
Boersma	Low	9	15									15	8			18	3
	High	9	25									11	13			20	2
	P	0.40										0.24				0.66	
Chin	Low	26	33	21	34	4						27	32	27	31	33	4
	High	28	30	26	28	5						16	43	14	34	38	4
	P	0.71		0.54								0.06		0.08		0.99	
Desmedt	Low	78	28	41	65	0	14	42	48	74	28	42	65				
	High	64	28	27	65	0	16	41	35	60	28	22	70				
	P	0.64		0.18			0.48			0.53		<0.05					
Farmer	Low											12	10				

	High											6	15				
	P											0.12					
Finak	Low	10	17	15	12	0	2	11	14	5	5	6	21	14	13	21	6
	High	11	15	16	9	1	1	12	13	3	6	4	22	12	14	22	4
	P	0.78		0.49			0.82			0.65		0.73		0.79		0.73	
Ginestier	Low	7	20	9	16	3	2	3	23			19	9	22	6		
	High	9	18	10	14	3	2	9	16			9	18	12	15		
	P	0.77		0.92			0.12					<0.05		<0.05			
Gluck	Low			1	41	34	8	25	37			39	36	51	24	56	20
	High			1	30	45	11	24	32			31	45	46	30	63	13
	P			0.20			0.67					0.19		0.40		0.24	
Hess	Low	27	40	8	35	24	1	23	43	3	7	34	33	43	22	48	18
	High	35	31	5	35	26	1	31	34	3	5	17	49	32	33	51	15
	P	0.17		0.68			0.33			0.71		<0.01		0.08		0.69	
Julka	Low			0	2	20	0	10	2			9	12	10	11	12	9
	High			0	3	19	1	12	2			12	10	13	9	12	10
	P			0.89			0.65					0.55		0.55		0.99	

Kreike	Low	26	0				8	6	12								
	High	23	0				2	6	15								
	P	0.99					0.15										
Loi	Low	2	42	22	21	1	7	18	10	37	4			13	30		
	High	1	42	21	21	1	10	19	6	31	10			8	34		
	P	0.99		0.99			0.46			0.14				0.32			
Loi 3	Low	3	36	17	22	0	7	10	13	32	6			12	27		
	High	3	35	17	20	1	7	10	11	34	4			6	32		
	P	0.99		0.58			0.95			0.74				0.18			
Lu	Low			26	35	4	5	10	50			38	27			44	21
	High			29	32	4	22	22	20			15	49			54	10
	P			0.86			<0.0001					<0.0001				<0.05	
Ma 3	Low	2	28	16	14	0	2	22	6	19	8	1	29	5	25	27	1
	High	0	30	14	15	1	1	17	12	18	8	0	30	5	25	25	2
	P	0.49		0.56			0.23			0.99		0.99		0.99		0.61	
Nikolsky	Low	39	31				3	29	30			31	39	38	32	45	25
	High	34	30				3	27	25			29	37	38	27	41	26

	P	0.86					0.95					0.99		0.73		0.73	
Radvanyi	Low	9	18	10	14	2	2	12	13								
	High	4	20	7	11	2	5	6	9								
	P	0.21		0.95			0.219										
Richardson 2	Low											14	5	15	4	15	3
	High											10	10	11	9	14	5
	P											0.19		0.18		0.69	
Schmidt	Low						17	64	19								
	High						12	72	16								
	P						0.45										
TCGA	Low	34	78	32	90	24						45	98	63	80	73	29
	High	30	62	30	85	26						15	122	34	102	81	22
	P	0.76		0.90								<0.0001		<0.01		0.26	
vandeVijver	Low									101	45	47	102				
	High									95	46	22	124				
	P									0.80		<0.001					
vantVeer	Low	44	15	30	29	0	2	9	48			23	36	29	30		

	High	38	20	37	20	1	10	18	30			16	42	20	38		
	P	0.32		0.18			<0.01					0.24		0.13			
Waddell	Low											14	19	16	14	19	6
	High											13	23	12	24	24	6
	P											0.63		0.14		0.75	
Wang	Low									84	52	41	102				
	High									96	41	36	107				
	P									0.16		0.59					
West	Low											17	8				
	High											7	17				
	P											<0.05					
Zhao	Low	7	19				2	17	8			11	12	11	11	16	6
	High	10	16				3	21	4			3	22	6	18	12	8
	P	0.56					0.38					<0.05		0.13		0.52	
Study count		0		0			2			0		8		2		1	
Misses		16		15			15			8		13		14		13	
Total studies		16		15			17			8		21		16		14	

% total	0	0	11.7	0	38.1	12.5	7.1
P	0.44	0.46	0.13	0.66	<0.0001	0.15	0.36

Low *vs.* high *SCN1B* expression cut-off is at 50th percentile. Study count, the number of datasets in which histoclinical characteristic associates with *SCN1B* expression. Misses, the number of datasets in which there was no association. % total, the % of datasets in which the histoclinical characteristic associates with *SCN1B* expression. P values within each dataset are from Fisher's exact tests except for pathological tumour size and grade, which are from χ^2 tests. P values for meta-analysis of association between *SCN1B* expression and individual histoclinical characteristics across multiple datasets, are from binomial tests.
