# Supplementary material for: The sodium channel β1 subunit mediates outgrowth of neurite-like processes on breast cancer cells and promotes tumour growth and metastasis
Source: Int J Cancer. 2014 Apr 12;135(10):2338–51. doi: 10.1002/ijc.28890 (PMC4200311; doi:10.1002/ijc.28890)
Supplement: Supplementary file 13 — Supplementary Information Table 3. Patient histoclinical characteristics and β1 expression. [file ijc0135-2338-SD8.pdf]

Table S3 Patient histoclinical characteristics and β1 expression.

Variable	β expression		P
	Low (%)	High (%)	
Age			
≤50	8 (12.3)	17 (26.2)	0.23
>50	7 (10.8)	33 (50.8)	
ER			
-	7 (10.6)	27 (40.9)	0.77
+	8 (12.1)	24 (36.4)	
Grade			
1	3 (4.5)	2 (3.0)	0.10
2	7 (10.6)	33 (50.0)	
3	5 (7.6)	16 (24.2)	
Menopausal status			
Premenopausal	7 (10.6)	21 (31.8)	0.77
Postmenopausal	8 (12.1)	30 (45.5)	
Node status			
-	7 (10.8)	20 (30.8)	0.77
+	8 (12.3)	30 (46.2)	
5-year BCa-specific survival			
Alive	11 (18.0)	47 (77.0)	0.11
Dead	2 (3.3)	1 (1.6)	

P values are from Fisher's exact tests except for grade, which is from χ^2 tests.
